# Clinical and immunological spectrum of five patients with activated phosphoinositide 3-kinase δ syndrome 1: A multicentric study from India

**DOI:** 10.70962/jhi.20250251

**Published:** 2026-04-21

**Authors:** Sagar Bhattad, Neha Singh, Rachna S. Mohite, Jyothi Janardhanan, Srikanta J.T., Anuj Shukla, Srinivas Jakka, Ranjit B. Joseph

**Affiliations:** 1 Paediatric Immunology, Rheumatology and Bone Marrow Transplant, Manipal Hospital, Bangalore, India; 2 Aster CMI Hospital, Bangalore, India; 3 Department of Paediatrics, ESIC Medical College and Hospital, Ranchi, India; 4 Paediatric Immunology and Rheumatology, Sai Child Care Hospital, Navi Mumbai, India; 5 Paediatric Immunology and Rheumatology, Aster MIMS, Kannur, India; 6 Department of Paediatric Pulmonology, Allergy and Sleep Medicine, Manipal Hospital, Bangalore, India; 7 Niruj Rheumatology Clinic, Ahmedabad, India; 8 Department of Paediatrics, Ankura Children’s Hospitals, Hyderabad, India; 9 Department of Paediatrics, Aster Medcity, Kochi, India

## Abstract

Activated phosphoinositide 3-kinase δ syndrome 1 (APDS1) is a combined immunodeficiency caused by monoallelic gain-of-function mutations in the *PIK3CD *gene. Patients with APDS1 have significant sinopulmonary involvement, lymphoproliferation, and autoimmune manifestations. We analyzed the clinical profile, treatment, and outcomes of five patients with APDS1. A total of 556 patients were diagnosed with inborn errors of immunity at our center between February 2017 and October 2025. Five patients had APDS1, confirmed by next-generation sequencing. Their records were analyzed in detail. The male-to-female ratio was 4:1. The mean age at symptom onset and at diagnosis was 14.8 and 62 mo, respectively. The age at onset of infections (mean age: 26 mo) preceded lymphoproliferation (mean age: 36 mo). Sinopulmonary infections (*n = 3), *recurrent gastroenteritis (*n* = 2), oral candidiasis (*n* = 1), and meningoencephalitis (*n* = 1) were the most commonly noted infections. Lymphoproliferation was seen in four patients, the most common being cervical lymph node enlargement (*n* = 4), followed by adenotonsillar hypertrophy (*n* = 3) and chronic splenomegaly (*n* = 3). Three of these patients with lymphoproliferation had Epstein-Barr viremia. Inflammatory colitis (*n* = 1) was the sole autoimmune manifestation noted in our cohort. Lymphocyte subset analysis showed increased CD8 cells and a reversal of the CD4:CD8 ratio (*n* = 4). Therapy included antibiotic prophylaxis (*n* = 5), intravenous immunoglobulin replacement therapy (*n* = 3), steroids (*n* = 3), mycophenolate mofetil (*n* = 1), and sirolimus (*n* = 1). Presently, four patients are alive and doing well, while one child is lost to follow-up. To the best of our knowledge, this is the first case series of APDS1 from the Indian subcontinent. We observed a significant delay in the diagnosis of these patients highlighting the need for raising awareness regarding this entity among pediatricians and primary care physicians.

## Teaser

Activated phosphoinositide 3-kinase δ syndrome 1 (APDS1) is a combined immunodeficiency caused by monoallelic gain-of-function mutations in the PIK3CD gene. 556 patients were diagnosed with inborn errors of immunity at our center between February 2017 and October 2025. Five patients had APDS1, confirmed by next-generation sequencing. Their records were analyzed in detail.

## Introduction

Activated phosphoinositide 3-kinase delta syndrome (APDS) is an inborn error of immunity (IEI) affecting humoral and cellular immunity. Angulo et al. first described it in 2013 in a cohort of 17 patients (1). A year later, Lucas et al. described a similar cohort marked by specific activating mutations in *PIK3CD* and referred to it as a p110δ-activating mutation causing senescent T cells, lymphadenopathy, and immunodeficiency (2). The underlying genetic defect in APDS is a monoallelic gain-of-function mutation in the *PIK3CD* (APDS1) and *PIK3R1* (APDS2) genes, which encode the catalytic p110δ subunit and the regulatory p85α subunit of phosphoinositide 3-kinase (PI3K), respectively (3). Clinical manifestations include sinopulmonary infections, lymphoproliferation, and heightened susceptibility to developing autoimmune complications and hematologic malignancies (4).

The PI3Ks regulate a wide range of biological functions across different tissues. Of these, the class I PI3Ks are mainly expressed in leukocytes and are essential for cell functions, including proliferation, growth, differentiation, and survival. A gain-of-function mutation activates downstream signaling by increasing the phosphorylation of Akt and mTOR. This has a profound effect on both the T and B lymphocyte lineages and on innate immune cells. T lymphocytes are prone to premature senescence and are characterized by an increase in CD8^+^ CD57^+^ cells. Impaired B lymphocyte maturation and class switching lead to elevated IgM and low IgG and IgA levels in a subset of these patients (2).

There is a dearth of literature regarding APDS from the Indian subcontinent. To address this lacuna, we aim to provide a comprehensive summary of the clinical and laboratory observations made in five APDS patients.

## Results

During the study period, five patients were diagnosed with APDS type 1. The male-to-female ratio was 4:1. Age at onset of symptoms was 14.8 mo (2, 30 mo), with a mean age at diagnosis of 5.2 years (11, 108 mo). Mean diagnostic delay was 47 mo (8, 60 mo). Age of onset of infections (mean age 26 mo) preceded lymphoproliferation (mean age 36 mo) in our cohort. Table 1 summarizes the clinical data of patients.

**Table 1.** Clinical profile of five APDS patients

​	P1	P2	P3	P4	P5
Sex	Male	Female	Male	Male	Male
Age at onset of infection (months)	3	3	24	30	14
Age at diagnosis (months)	60	11	84	48	108
Diagnostic delay	57	8	60	18	94
Age at onset of lymphoproliferation (months)	3	-	60	36	48
Lymphoproliferation	Cervical, submandibular, and axillary lymphadenopathy, adenotonsillar hypertrophy, splenomegaly	-	Cervical and mediastinal lymphadenopathy, follicular bronchitis, hepatosplenomegaly	Cervical, axillary, mediastinal, and mesenteric lymphadenopathy, adenotonsillar hypertrophy (Fig. 2 a)	Cervical lymphadenopathy, adenotonsillar hypertrophy
Mean age of onset of infections^a^ (months)	36	9	36	-	24
Sinopulmonary infections	-	Yes	Yes (Fig. 2 b)	-	Yes
Other infections	Viral exanthematous fever	-	Recurrent gastroenteritis	Oral candidiasis	Recurrent gastroenteritis, meningoencephalitis
Organisms	EBV, varicella	-	EBV	EBV, *Candida tropicalis*	-
Histopathology	**Cervical lymph node biopsy:** marked interfollicular paracortical zone expansion by lymphoid cells with small cleaved and intersperse immunoblasts and increase in paracortical high endothelial venules and sclerosis. The expanded areas showed atrophic lymphoid follicles. Large follicles with reactive germinal center with indistinct mantle zones	NA	**Bronchial lymph node biopsy:** lymphoid aggregates in subepithelial stroma, lymphocytic exocytosis in mucosal lining. Stroma showed plasma cells entrapping mucosal glands (Fig. 2)	**Axillary lymph node biopsy:** cortex containing secondary follicles with prominent germinal centers. Paracortex expanded and showed small-to-medium lymphocytes, histiocytes, high endothelial venules, and plasma cells	NA
Autoimmunity	​	VEO-IBD	-	-	​
Other	-	Developmental delay, oral aphthosis	-	-	Developmental delay
Treatment	SteroidsMycophenolate mofetil (MMF) Sirolimus Antibiotics	Monthly IVIG, antibiotics	Monthly IVIG, steroids, antibiotics	Monthly IVIG, antibiotics, steroids	Antibiotics
Outcome	Alive	Alive	Lost to follow-up	Alive	Alive

EBV, Epstein-Barr virus; VEO-IBD, very early-onset inflammatory bowel disease.

a Only systemic or persistent infections were included while calculating the mean age of onset of infections. Oral candidiasis was excluded.

Rhinosinusitis (*n* = 3), otitis media (*n* = 2), and pneumonia (*n* = 2) were noted in three patients (P2, P3, and P5). Other infections included recurrent gastroenteritis (P3, P5), oral candidiasis (P4), and meningoencephalitis (P5).

**Figure 1.**
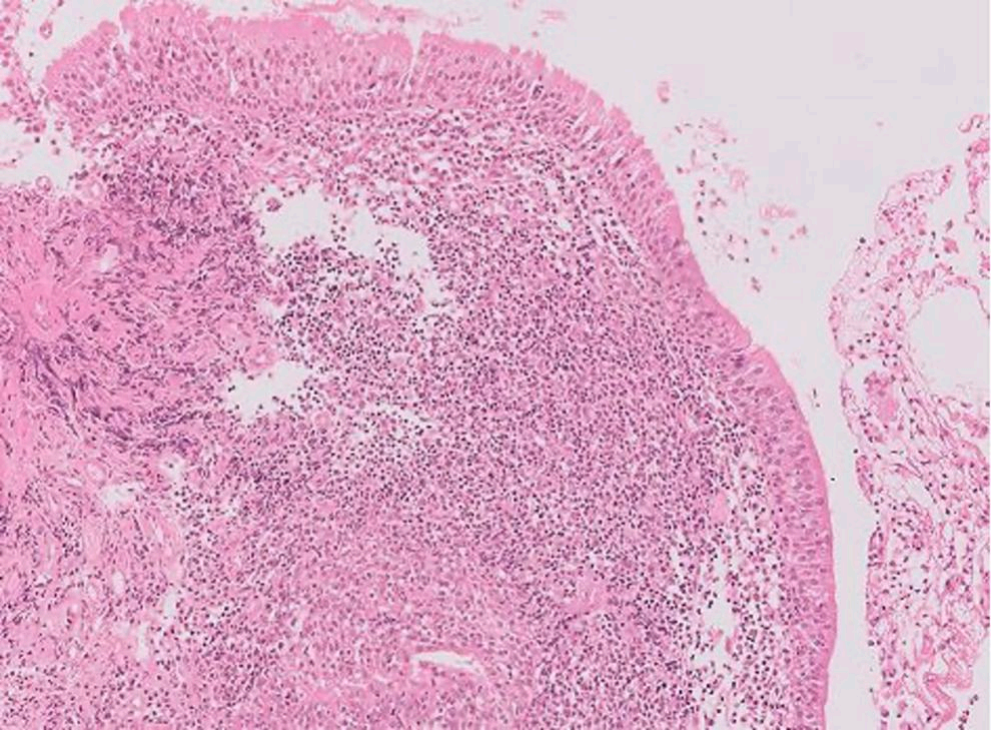
**Photomicrograph (H&E original magnification ×400) showed lymphoid aggregates in subepithelial stroma.** Lymphocytic exocytosis in the mucosal lining was noted. Stroma showed plasma cells entrapping mucosal glands.

**Figure 2.**
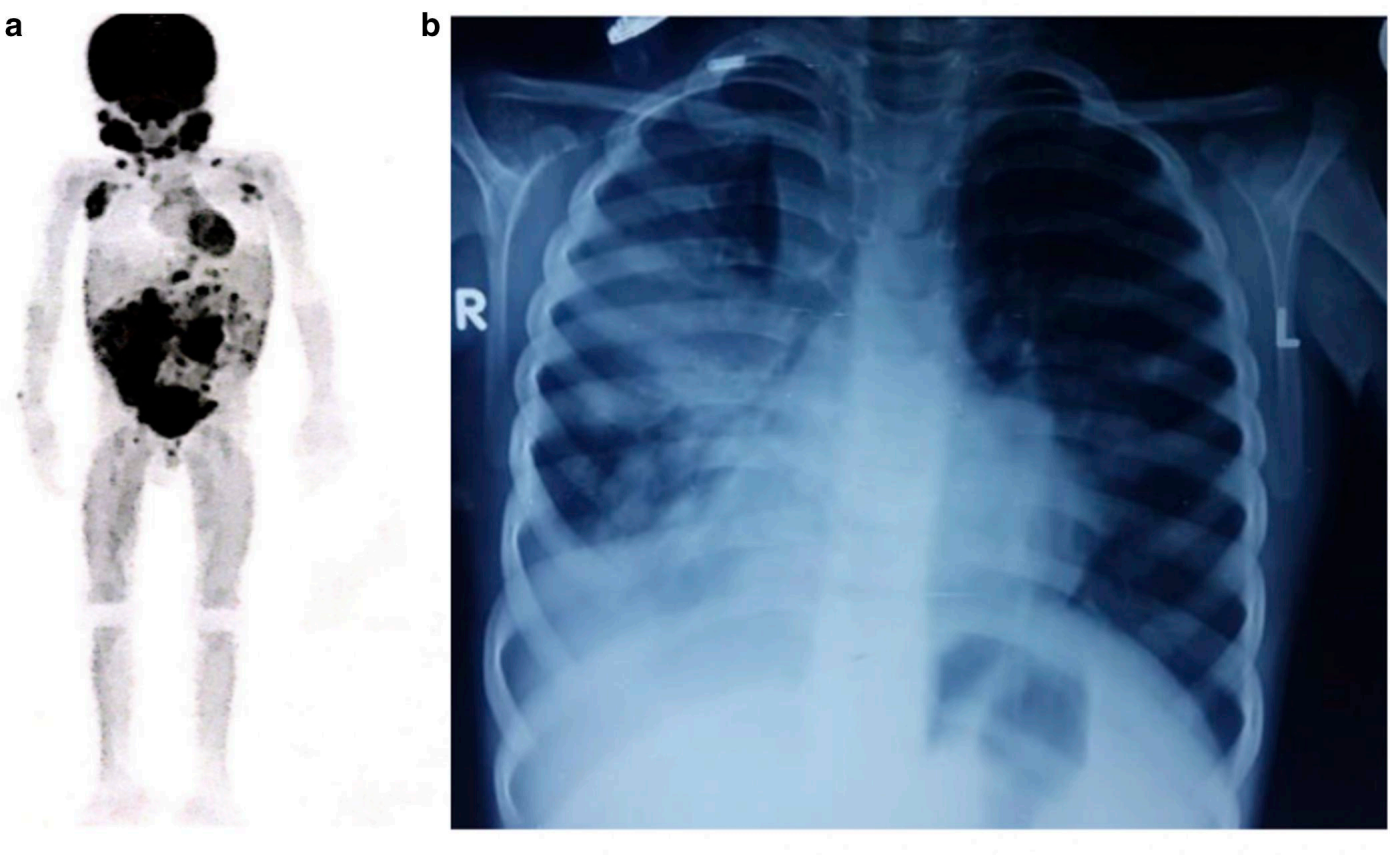
**(a)** Fluorodeoxyglucose positron emission tomography of P4 showed multiple enhancing enlarged discrete conglomerated supra- and infradiaphragmatic lymph nodes, bulky adenoids, bilateral tonsils, and ground glass nodules in bilateral lung parenchyma. **(b)** Chest radiograph of P3 showed right lobar pneumonia.

Lymphoproliferation was documented in four patients. Cervical lymphadenopathy (*n* = 4) was most common, followed by axillary (*n* = 2), mediastinal (*n* = 2), and mesenteric lymphadenopathy (*n* = 1). Adenotonsillar hypertrophy was a persistent finding in three patients. Chronic splenomegaly was seen in three patients, while one patient also had hepatomegaly. Interestingly, significant nodularity was noted over the posterior membranous wall throughout the tracheobronchial tree in P3 during bronchoscopic evaluation. Lymphoproliferation was triggered by Epstein-Barr virus (EBV) infections in three patients. P3 had high EBV viral load in the blood, while EBV stain was positive on lymph node biopsy in P1. P1 and P4 were positive for EBV VCA antibody. Histopathology of the biopsied lymph nodes was available for three patients. Lymph node biopsies showed reactive germinal centers with paracortical zone expansion, multiple lymphocytes, histiocytes, plasma cells, and high endothelial venules (P1, P3). Bronchial tissue biopsy from P3 showed lymphoid aggregates in subepithelial stroma, lymphocytic exocytosis in mucosal lining, and plasma cells entrapping the mucosal glands, suggestive of follicular hyperplasia (Figs. 1 and 2).

Very early-onset inflammatory bowel disease (VEO-IBD) was the only autoimmune manifestation seen in one patient. P2 presented with chronic gastroenteritis, oral aphthosis, and failure to thrive since 3 mo of age. On evaluation, she had high fecal calprotectin levels (969.4 microg/g) and hence was diagnosed with inflammatory bowel disease.

Immunological data for patients at the time of evaluation are shown in Table 2. P1 and P3 had elevated IgG levels, while P2 had elevated IgA levels. Three patients (P1, P3, and P5) had elevated IgM levels at the time of diagnosis. Lymphocyte subsets were available for four patients, who showed elevated CD8 cells and a reversal of the CD4:CD8 ratio. On exome sequencing, four patients (P1, P3, P4, and P5) had previously reported missense mutations, while P2 had a de novo frameshift insertion (American College of Medical Genetics [ACMG] criteria PM2, PS2, PVS1, PP5, PP4) in the *PIK3CD* gene. The pathogenicity was subsequently established by performing Sanger sequencing and segregation analysis.

**Table 2.** Laboratory workup of the five patients

​	P1 (5 mo)	P2 (11 mo)	P3 (7 years)	P4 (4 years)	P5 (9 years)
IgG (mg/dl) (minimum–maximum)	**1,100** (176–601)	843 (172–1,069)	**1,830** (608–1,572)	931 (345–1,236)	930 (608–1,572)
IgA (mg/dl) (minimum–maximum)	31 (4.4–84)	**332** (11–106)	138 (33–236)	50 (14–159)	66 (33–236)
IgM (mg/dl) (minimum—maximum)	**298** (17–105)	95 (41–173)	**233** (43–207)	146 (43–207)	**433** (52–242)
IgE (IU/ml)	-	6.8	41.6	-	13.8
Lymphocyte subset assay					
CD3	-	70% (3,254)	67.2% (1,927)	67% (1,370)	86% (4,078)
CD19	-	8.1% (375)	3.6% (104)	13% (288)	7% (332)
CD56	-	18.7% (861)	28.9% (829)	18% (386)	6.5% (309)
CD4	-	18.7% (861)	17.8% (512)	27% (556)	11% (453)
CD8	-	35% (1,652)	40.4% (1,160)	38% (777)	86% (3,491)
CD4/CD8	-	**0.75**	**0.44**	**0.72**	**0.13**
Whole-exome sequencing					
Gene	*PIK3CD*				
Mutation position	c.3061G>A (p.Glu1021Lys)	c.1332_1333insTGGGCCTG (p.Val445fs)	c.1573G>A (p.Glu525Lys)	c.3061G>A (p.Glu1021Lys)	c.3061G>A (p.Glu1021Lys)
Exon	Exon 24	Exon 10	Exon 10	Exon 24	Exon 24
Zygosity	Heterozygous	Heterozygous	Heterozygous	Heterozygous	Heterozygous
Pathogenicity	Pathogenic	Pathogenic	Pathogenic	Pathogenic	Pathogenic
Reported	Refs. 1, 2, 5, 6, 7, 8	Novel (ACMG criteria PM2, PS2, PVS1, PP5, PP4)	Ref. 2	Refs. 1, 2, 5, 6, 7, 8	Refs. 1, 2, 5, 6, 7, 8

Therapy included antibiotic prophylaxis (*n* = 5), intravenous immunoglobulin replacement therapy (IVIG) (*n* = 3), steroids (*n* = 3), mycophenolate mofetil (*n* = 1), and sirolimus (*n* = 1). Four patients are alive and doing well, while P3 is lost to follow-up.

## Discussion

APDS is a combined immunodeficiency with a Common Variable Immune Deficiency (CVID)-like phenotype. Our patients had an early age of diagnosis at 5.2 years, with a mean diagnostic delay of ∼4 years. Severe infections and lymphoproliferation were seen in four patients each (Table 1). In contrast, the largest cohort of APDS patients described by Coulter et al. (*n* = 53) had a mean age of 17 years. Although most of the patients were of European descent (*n* = 42), two Indian patients were also included in the study. The most common presentation of these patients was recurrent respiratory infection (51/53), with age of onset ranging from 1 to 7 years (4).

Coulter et al. reported pneumonia as the most common respiratory finding, followed by bronchiectasis and recurrent otitis media. Bacterial infections were common, and *Streptococcus pneumoniae* and *Haemophilus influenzae* were the two most commonly isolated pathogens in these patients. Infections with the herpesvirus group were more frequent in these patients (4). Lucas et al. reported that all 9 patients had EBV viremia, while Angulo et al. reported herpetic infections in 4 of 17 (24%) of their cohort (1, 2). The findings were different in our cohort: the most common finding was upper respiratory tract infections (*n* = 3), followed by otitis media (*n* = 2) and pneumonia (*n* = 2). 60% (3/5) of our patients had viral infections. None of our patients had developed bronchiectasis at the time the manuscript was prepared. This could possibly be related to younger age of our cohort, with a mean age of 5.2 years.

Coulter et al. reported lymphoproliferation as the second most common manifestation, affecting 75% of their 53 APDS patients. Lymphadenopathy (*n* = 34) was the most common manifestation, followed by splenomegaly (*n* = 31) and hepatomegaly (*n* = 24) (4). Cervical lymph nodes (*n* = 14) were the most frequently affected group. The majority of their patients had concurrent EBV and CMV infections. Tonsillitis was another prominent manifestation reported in 15 patients, with seven undergoing tonsillectomy. This was similar to our cohort, where all four patients had cervical lymphadenopathy and three had adenotonsillar hypertrophy. Autoimmunity is well reported in APDS and is known to affect one third of patients. In our cohort, VEO-IBD was the sole autoimmune manifestation (4). Interestingly, 9 of 13 patients with gastrointestinal involvement in the study by Coulter et al. had nodular mucosal lymphoid hyperplasia on histopathology (4). The VEO-IBD in our patient could be a part of lymphoproliferation. Unfortunately, we did not have any biopsy evidence to confirm it.

On histopathology, lymph nodes in APDS patients showed follicular hyperplasia with an atrophic mantle zone. Germinal centers were frequently invaded by follicular T cells (PD1^+^, CD57^+^). There was parasinusoidal B cell aggregation with a reduction in IgG-producing plasma cells. The mucosa-associated lymphoid tissue in the gastrointestinal and respiratory tracts also underwent nodular lymphoid hyperplasia (4). The lymph node biopsy of our patients showed similar findings, with paracortical expansion suggestive of T cell expansion. The bronchial tissue biopsy from P3 showed classical lymphoid aggregates in subepithelial stroma, and lymphocytic exocytosis in the mucosal lining, suggestive of follicular hyperplasia.

APDS1 patients are also known to have a hyper-IgM–like phenotype with elevated IgM levels with low IgG levels (9). Low IgA levels were reported in these patients. Three patients in the cohort by Coulter et al. had normal IgG but low IgG2 levels (4). Our cohort had three patients with elevated IgM levels, one patient had elevated IgA levels and two patients had elevated IgG levels. APDS1 is characterized by increased senescent CD8 T cells (CD8 CD57^+^), CD4:CD8 reversal, and raised HLA-DR^+^ expression both on CD4^+^ and on CD8^+^ T cells, suggestive of the activated state of these cells (9). Lymphocyte subset analysis showed a CD4:CD8 reversal in four patients in our cohort.

The genetic mutations described in APDS1 patients are germline monoallelic. E1021K was the most commonly identified mutation, followed by E525K. Other described mutations in APDS1 include N334K and C416R (4). Four patients had previously reported mutations, while one patient had a novel frameshift insertion mutation.

The available treatment options in APDS patients include IVIG, broad-spectrum antibiotics, antifungals, and antiviral drugs. The availability of targeted therapy in the form of rapamycin (mTOR) inhibitors like sirolimus and newly described PI3K inhibitors, including leniolisib and seletalisib, has improved the prognosis and outcome of these patients (10). Hematopoietic stem cell transplant is an option for refractory cases (11). The two case series of Hematopoietic Stem Cell Transplant (HSCT) in APDS patients showed promising results with the survival of 9/11 and 7/9 patients (12, 13). In our cohort, antibiotic prophylaxis and IVIG were used for treatment in five and three patients, respectively, while three of them required steroids, mycophenolate mofetil, and sirolimus.

We acknowledge our limitations. We did not quantify senescent T cells (CD4+CD57+ and CD8+CD57+ T cells) and could not perform PI3K-Akt-mTOR functional assay due to the lack of availability of such testing facilities.

## Materials and methods

Our hospital is a tertiary care center in South India that is actively involved in diagnosing and treating patients with IEI. During the study period from February 2017 to October 2025, 556 patients with various IEIs were diagnosed at our center. Five patients with APDS1 were included in the current study. These patients were referred from various centers across India. Data on demographics, clinical manifestations, laboratory features, therapeutic management, and outcomes were collected retrospectively from the medical records. The study was approved by the Institutional Ethics Committee (IEC No. ECR/1084/Inst/KA/2018).

Serum immunoglobulin levels were measured using a nephelometer (Siemens Atellica NEPH 630). The immunological analysis involved characterizing lymphocyte subsets. The markers used were CD3, CD4, CD8 for T cells, CD19 for B cells, and CD56 DC16 for natural killer cells. This was achieved through a commercial kit (Multitest 8-Color TBNK Reagent, BD Biosciences). Strict quality control was maintained using internal and external controls. Genetic evaluation was performed through whole-exome sequencing on an Illumina NovaSeq 6000 platform, capturing the protein-coding genome regions with a customized kit. Data analysis employed established tools such as BWA-MEM, Picard tools, GATK, and Annovar, aligning sequences to reference data from National Center for Biotechnology Information RefSeq and human genome build GRCh37/UCSC hg19. Stringent filtering criteria included a minimum read depth of 10X and a Phred score exceeding 30. Relevance to the clinical context was ensured by integrating human phenotype ontology terms. The ACMG criteria were employed to categorize observed genetic variants.

The data on immunoglobulin and absolute lymphocyte counts were coded and entered into the IBM Statistical Package for Social Sciences for Windows, version 25.0: IBM Corp. Descriptive statistics were used to calculate the mean age of onset, diagnosis, and delay.

### Conclusion

There was a significant delay in the diagnosis of the APDS patients in our cohort underlining the need for raising awareness about this disease. The non-malignant lymphoproliferation was viral-driven in most cases, and one must screen for EBV in this setting. Sirolimus is a useful agent in the treatment of lymphoproliferation in APDS patients, especially in resource-constrained settings where targeted therapies are not easily available.
